# Teaching in Paediatrics for UK Foundation Doctors: A Cross-Sectional Study

**DOI:** 10.7759/cureus.47714

**Published:** 2023-10-26

**Authors:** Emma C Alexander, Hrisheekesh J Vaidya, Charlotte Burford, Roshni Mansfield

**Affiliations:** 1 Paediatrics, Queen Mary University of London, London, GBR; 2 Critical Care, University College London, London, GBR; 3 Surgery, East Kent Hospitals University NHS Foundation Trust, London, GBR; 4 Paediatrics, University of Oxford, Oxford, GBR

**Keywords:** postgraduate, specialism, foundation programme, paediatrics, training

## Abstract

Background

Most doctors will care for children regularly during their careers in settings such as the emergency department, general practice, surgery, or, for a minority, during paediatric specialist training. As such, exposure to topics related to child health ought to be part of the broad curriculum of learning offered to UK Foundation Programme doctors.

Objective

This study aimed to quantify teaching in paediatrics that is accessed by Foundation doctors.

Methods

A cross-sectional electronic survey of foundation year one or two (F1/F2) doctors at the end of the 2020-2021 academic year. Ethical approval was granted by the Imperial College London (ICL) Education Ethics Review Process (EERP 2021-082).

Results

Two-hundred and five Foundation doctors completed the survey, from 16 of the 18 Foundation schools. Respondents attended a median of two hours (interquartile range (IQR) 0-10) of paediatric teaching during the past 12 months, including a median of one hour (IQR 0-2) of core teaching and a median of one hour (IQR 0-9) of non-core teaching. Those who had worked in a paediatric post in the past 12 months, or who were interested in Paediatrics as a career, attended more median hours of teaching.

Conclusions

Although many doctors will care for children routinely during their later careers, the number of teaching hours in paediatrics experienced by Foundation doctors is low. The UK Foundation Programme should incorporate more teaching in paediatrics to increase exposure to child health amongst newly graduated and as-yet unspecialised doctors.

## Introduction

The UK Foundation Programme is a two-year training programme that aims to provide newly qualified doctors with sufficient exposure and experience across key specialties, such that they have the requisite knowledge and skills to enter specialty or general practitioner (GP) training [1]. This usually comprises six four-month posts. Typically, trainees must do at least one post in medicine, surgery, a community placement (most commonly GP) and acute/emergency medicine; other subspecialties comprise the remainder of the two-year full-time-equivalent training period. Completion of the Foundation Programme is mandatory for UK medical graduates, in order for them to apply for further specialist or general practice training. 

A minority of Foundation doctors work in a paediatric post during the UK Foundation Programme (e.g. 8% of foundation year one (F1s) and 16% of foundation year two (F2s) in 2016) [2]. Consequently, working with children is not mandatory for progression into specialist or GP training. However, most doctors will care for children at some point during their careers, irrespective of any final specialisation, considering that routine treatment of children is common across general practice (with GP training posts representing 47.2% of 7911 eligible training posts in 2021), emergency medicine (4.0%) and paediatrics (4.7%) [3]. Anaesthetic trainees (5.7% of posts) typically complete training in paediatric anaesthesia, and most surgical trainees (6.3%) will also operate on children and adolescents during their training. However, despite this high likelihood of exposure during their careers, non-paediatric trainees have described a 'training gap' in paediatric issues and lack of confidence in managing children and young adults. For example, in a 2017 study of adult medical specialists, 70% rated their training in adolescent and young adult health as minimal or non-existent [4].

Foundation doctors are provided with a 'core' teaching programme, which typically involves one to two teaching hours per week delivered by their employing hospitals on important areas of clinical knowledge and skills. Every year, F1s and F2s must log their attendance at a minimum of 30 'core' teaching hours and 60 teaching hours in total to pass their Annual Review of Competency Progression (ARCP) (this was relaxed to 45 teaching hours of any kind during the 2020-2021 academic year due to the pressures of the COVID-19 pandemic) [5]. The core teaching curriculum is not centrally administered or mandated, but it can represent the only opportunity for formal training in paediatrics before sub-specialisation. Despite the potential availability of teaching in paediatrics in these sessions, Foundation doctors are rarely able to attend every session, due to night shifts, scheduled days off or high clinical commitments that prevent them from leaving the clinical environment for short periods [6,7]. As a result, it is not uncommon for Foundation doctors to engage in voluntary educational activities, including e-learning, courses, higher qualifications and attendance at conferences, which may provide further opportunities for interested trainees to increase their exposure to teaching in paediatrics [8]. Foundation doctors can also arrange ‘taster weeks’ in a specialty of their choice, but exposure to paediatrics in this environment would depend on a trainee having a pre-existing interest [9]. Therefore, although not mandating a formal list of topics, the core teaching programme represents the only avenue of teaching that is theoretically offered to all trainees, irrespective of any prior interest in paediatrics.

Thus far, despite the importance of experience and knowledge of this patient group for most doctors’ careers, no study has attempted to quantitatively evaluate the amount of teaching Foundation doctors receive in paediatrics, including core teaching and teaching from other sources. This study seeks to quantify the amount of teaching in paediatrics that was offered to Foundation doctors during the 2020-2021 academic year. 

This article was previously presented as a poster at the Royal College of Paediatrics and Child Health (RCPCH) Conference 2022 [10].

## Materials and methods

A cross-sectional survey of junior doctors working at the F1 or F2 level at hospitals in the UK was performed. All F1 and F2 doctors (a national total of around 16,000 at any one time) were eligible for inclusion. The survey was first drafted by the authors, with this draft reviewed with feedback from the Paediatric Educators Special Interest Group (PEdSIG) committee, a national organisation of paediatric professionals with expertise in paediatric education, before finalisation. As a simple survey using count data and Likert scales, with no previous similar report in the literature, no formal template was used to inform its creation.

The electronic survey was available for completion for two months between the 30th of May 2021 and the 4th of August 2021. The survey was distributed to potential participants by the following methods: 1) The lead author contacted local postgraduate education teams across the UK and requested they distribute the survey to their regional Foundation doctor mailing lists. 2) Regional representatives from the UK Aspiring Paediatricians Society (UKAPS) advertised the survey locally. 3) The UKAPS and PEdSIG advertised the survey on Twitter.

The survey collected information on the participants’ type of degree, training location, experience in paediatrics, interest in paediatrics, type of teaching participated in (checkbox), a quantitative estimate from participant of number of hours spent on each type of teaching over the past 12 months, experience of child protection/safeguarding teaching and a Likert scale regarding the adequacy and importance of teaching in paediatrics.

Foundation doctors were informed at the time of their voluntary participation that the completion of the survey indicated consent to participate and were informed that the results of the survey may be shared through academic publication. Ethical approval was granted by the Imperial College London Education Ethics Review Process (EERP) (EERP 2021-082).

Statistical analysis

All analyses were conducted using IBM SPSS Statistics for Windows, versions 27 and 28 (released 2020 and 2021; IBM Corp., Armonk, New York, United States). Data were manually cleaned and the mid-points of duration ranges were determined (e.g. if a participant wrote an estimate of five to 10 teaching hours attended, this was averaged to 7.5 hours). Participant interest in a career in paediatrics was defined by their response to the question 'Are you interested in applying for paediatric specialist training (ST1)?' and if they also confirmed that they had either submitted an application for specialist training or that they were going to in the future. A Shapiro-Wilk test for normality confirmed that data on the number of teaching hours were non-normally distributed for all subcategories of participants. Data were therefore analysed using non-parametric statistical testing methods (Mann-Whitney U for comparing continuous or ordinal variables between two discrete groups; chi-square analysis for comparing binary outcomes between two discrete groups). All respondents answered all the questions, so there were no missing data. A p-value <0.05 was considered significant for all analyses.

## Results

Participant characteristics

Overall, 205 Foundation doctors completed the survey. This was comprised of 49.8% (n=102) F1 doctors and 50.2% (n=103) F2 doctors (see Table 1). Overall, 75.1% (n=154) of the respondents completed an undergraduate medical degree straight from school, 15.6% (n=32) completed a graduate-entry medical degree, 8.3% (n=17) completed an undergraduate medical degree as a second degree and 1.0% (n=2) completed an extended medical degree programme. There were respondents from 16 of the 18 Foundation deaneries in the UK, with the greatest proportions of responders coming from Yorkshire and the Humber (n=34, 16.6%), North West England (n=27, 13.2%) and South Thames (n=27, 13.2%) and the fewest from West Midlands Central and Northern Ireland (n=1, 0.5% each). The Leicestershire, Northamptonshire and Rutland (LNR) and Northern deaneries were not represented.

**Table 1:** Participant characteristics, experience in paediatrics, interest status and type of teaching

Detail	Number (percentage)
Foundation year	
Foundation year 1	102 (49.8%)
Foundation year 2	103 (50.2%)
Type of medical degree	
Undergraduate degree (school leaver)	154 (75.1%)
Graduate-entry degree	32 (15.6%)
Undergraduate degree (post-degree)	17 (8.3%)
Extended medical degree programme	2 (1.0%)
Deanery	
East Anglia	11 (5.4%)
Essex, Bedfordshire and Hertfordshire (EBH)	2 (1.0%)
Leicester, Northamptonshire and Rutland (LNR)	0 (0.0%)
North Central and East London	12 (5.9%)
North West London	14 (6.8%)
North West England	27 (13.2%)
Northern	0 (0.0%)
Northern Ireland	1 (0.5%)
Oxford	10 (4.9%)
Peninsula	11 (5.4%)
Scotland	11 (5.4%)
South Thames	27 (13.2%)
Trent	16 (7.8%)
Wales	2 (1.0%)
Wessex	2 (1.0%)
West Midlands Central	1 (0.5%)
West Midlands North	6 (2.9%)
West Midlands South	4 (2.0%)
Yorkshire and Humber	34 (16.6%)
Experience in paediatrics	
Paediatric post in the past 12 months	50 (24.4%)
No paediatric post in the past 12 months	155 (75.6%)
Non-paediatric post in the past 12 months involving routine care of paediatric patients	93 (45.4%)
No non-paediatric post in the past 12 months involving routine care of paediatric patients	112 (54.6%)
Self-declared interest in paediatric training	
Yes, and I submitted an application this year	7 (3.4%)
Yes, and I am planning to submit an application in the future	33 (16.1%)
No	141 (68.8%)
Unsure	24 (11.7%)
Type of paediatric teaching accessed (multiple choice)	
Mandatory 'core' Foundation teaching	98 (47.8%)
Non-core teaching	
Online or in-person lectures	57 (27.8%)
Bedside teaching	32 (15.6%)
e-learning resources	50 (24.4%)
Simulation sessions	43 (21.0%)
Conferences	13 (6.3%)
Optional courses	18 (8.8%)
Non-mandatory teaching, not otherwise specified	60 (29.3%)
None/not applicable	61 (29.8%)
Child safeguarding teaching	
Yes	174 (84.9%)
No	31 (15.1%)

Teaching attended

The overall cohort of participants reported attending a median of two hours (interquartile range (IQR) 0-10) of paediatric teaching, in total, during the past 12 months. This comprised of a median of one hour (IQR 0-2) of 'core' Foundation teaching on paediatric topics over the past 12 months and a median of one hour (IQR 0-9) of non-core informal teaching.

**Figure 1:**
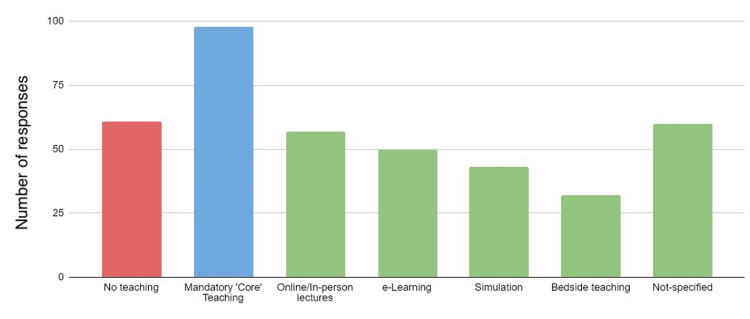
Bar chart showing the number of participants attending different types of teaching activity during the past 12 months. Green bars indicate non-core teaching activities, blue is core teaching, and red is no teaching.

When asked to state in a checkbox the different types of teaching attended, 47.8% (n=98) described attending 'core' Foundation teaching in paediatrics. Non-core types of teaching included online or in-person lectures (27.8%, n=57), e-learning resources (24.4%, n=50), simulation sessions (21.0%; n=43) and bedside teaching (15.6%; n=32). A further 29.3% (n=60) described attending non-core teaching but did not specify the form of teaching. A proportion (29.8%; n=61) described receiving no teaching of any form (Figure 1).

The completion of child safeguarding training is considered a mandatory requirement for ARCPs, which should be completed annually by all F1/F2s. Overall, 84.9% (n=174) reported receiving teaching in child safeguarding during F1/F2 (including e-learning), and 15.1% (n=31) reported not receiving any safeguarding teaching, of which 22 were F1 doctors and nine were F2 doctors.

Experience in paediatrics

Considering working exposure to children and young people over the past 12 months, overall, 24.4% (n=50) had completed a paediatric post during the past 12 months, and 45.4% (n=93) had completed a post (not including paediatrics) in which they had been involved in the routine care of paediatric patients during the past 12 months (e.g. GP or accident and emergency (A&E)). Almost half (44.3%; n=91) had neither completed a post in paediatrics nor completed another post involving the routine care of paediatric patients during this time.

Teaching attended by paediatric experience

**Figure 2:**
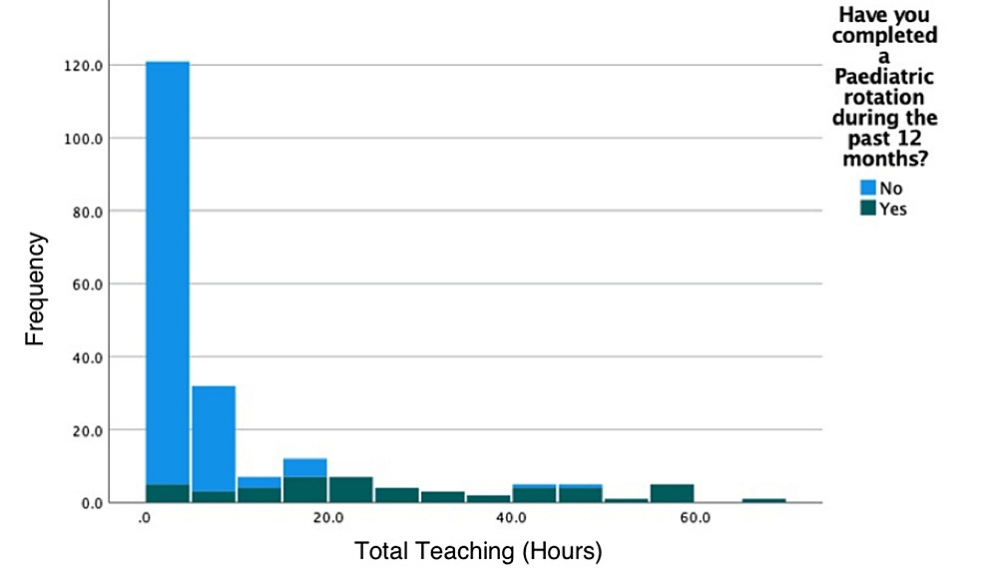
Stacked histogram of total teaching hours according to whether participants had completed a paediatric post during the past 12 months

The participants were split according to whether they had worked in a paediatric post during the past 12 months, either as an F1 or F2. The median number of total hours attended by those who had worked in a paediatric post was statistically significantly higher than in those who had not (median 24.25 hours vs. one hour, Mann-Whitney U, p<0.001; Figure 2).

Those who had worked in a paediatric post also reported attending significantly more core Foundation teaching on paediatric topics and more non-core teaching and missing more teaching on paediatric topics due to other commitments (Table 2). 

**Table 2:** Total and sub-types of paediatric teaching attended split by whether participants had worked in a paediatric post

	All (n=205)	Worked in paediatric post, 24.4% (n=50)	Did not work in paediatric post, 75.6% (n=155)	Worked in post vs did not work in post
	Median hours (IQR)			p-value
All teaching attended	2 (0-10)	24.25 (14.75-42)	1 (0-5)	<0.001
Mandatory ('core') Foundation teaching	1 (0-2)	2 (0-4)	0 (0-1.5)	0.001
Non-core teaching	1 (0-9)	20 (10-40)	0 (0-2)	<0.001
Hours not able to attend, due to work/other commitments (not included in ‘All teaching attended’)	0 (0-1.75)	6 (0.75-10)	0 (0-0)	<0.001

Teaching attended by pre-existing interest in paediatrics

Overall, when asked whether they were interested in applying for paediatric specialist training, 68.8% (n=141) said ‘No’. A total of 16.1% (n=33) respondents said ‘Yes, and I am planning to submit an application in the future’, 3.4% (n=7) said ‘Yes, and I submitted an application this year’ and 11.7% (n=24) were ‘Unsure’. When asked to indicate which other specialties respondents were interested in (across all specialties offered at the specialty training year one (ST1) and core training year one (CT1) entrance level), the most popular specialties for respondents were internal medicine by 33.7% (n=69), general practice by 30.7% (n=63) and acute care common stem (ACCS) anaesthetics/core anaesthetics by 23.4% (n=48).

**Figure 3:**
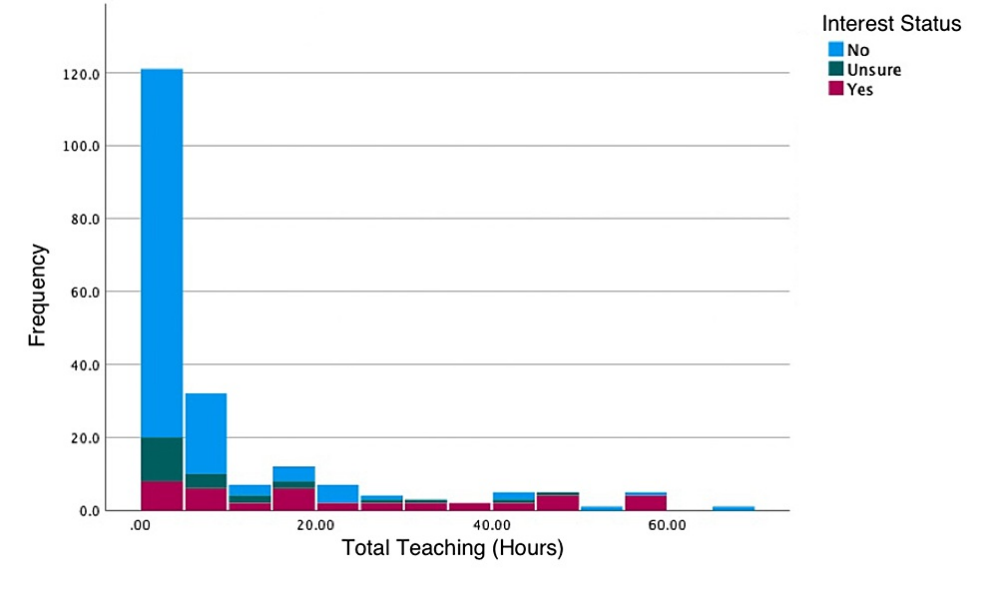
Stacked histogram of total teaching hours according to whether participants declared an interest in paediatrics

The median number of hours attended by those who were interested in paediatrics as a career was statistically significantly higher than in those who were not interested (median 18.25 hours (IQR 6-40) vs. two hours (IQR 0-6), Mann-Whitney U, p<0.001; Table 3, Figure 3).

**Table 3:** Total and sub-types of paediatric teaching attended split by whether participants were interested in paediatric training

	All (n=205)	Interested in paediatrics, 22.1% (n=40)	Not interested in paediatrics, 77.9% (n=141)	Interested vs. not interested in paediatrics
	Median hours (IQR)			p-value
All teaching attended	2 (0-10)	18.25 (6-40)	2 (0-6)	<0.001
Mandatory ('core') Foundation teaching	1 (0-2)	1 (0-3)	0.5 (0-2)	0.026
Non-core teaching activities	1 (0-9)	10 (0-30)	0 (0-6)	<0.001
Hours not able to attend, due to work/other commitments (not included in ‘All teaching attended’)	0 (0-1.75)	0 (0-5)	0 (0-1)	0.034

Those who were interested in paediatrics also reported attending significantly more core Foundation teaching on paediatric topics and more non-core teaching (Table 3).

Additional learning opportunities pursued by those interested in paediatrics

Considering only those participants who were interested in paediatrics (n=40), the median time spent attending learning opportunities outside the clinical environment or departmental teaching (excluding both core teaching and non-core teaching), for example, away on courses and conferences, was six hours (IQR 2-10). This was not significantly different between those who completed a paediatric post (six hours, IQR 3.5-10) compared to those who did not (six hours IQR 0-12, p=0.708).

Core teaching topics in paediatrics

For the participants who indicated that they had only undertaken mandatory 'core' teaching and provided examples of the sessions attended, the following topics were covered: common paediatric problems in general practice (n=5), assessment of the sick child (n=5), asthma (n=5), allergy (n=4), child safeguarding (n=4), paediatric surgery (n=2), paediatric radiology (n=1) and how to perform a baby check (n=1).

Opinion regarding provisions of paediatric teaching

Finally, the participants were asked whether they believed that teaching in paediatrics is adequate, and whether teaching in paediatrics was important. Across all the respondents, from a scale of 1-5 (1 = strongly disagree, 5 = strongly agree), the median Likert score in response to 'I believe that the level of paediatric teaching during the Foundation years is adequate' was 2 (disagree), and IQR was 1-3. When asked if 'I believe it is important for teaching in paediatrics to be offered to Foundation Doctors', the median Likert score was 5 (strongly agree) (IQR 4-5) for all the respondents, and there was no statistically significant difference between those who were interested in paediatrics to those who were not (median 4 (IQR 4-5) vs. median 4 (IQR 4-5); p=0.648).

## Discussion

In this study, we have described for the first time the provision of paediatric teaching received by Foundation doctors training across the UK. Our results describe minimal exposure to core teaching in paediatrics. Trainees working in individual posts in paediatrics experience substantially more teaching, as do those with an interest in paediatrics. Across all the respondents, the median number of hours of paediatrics teaching attended across a year was two hours, and the majority of participants did not feel that paediatrics teaching during the UK Foundation Programme was adequate. Foundation doctors are offered around one to two hours of core teaching per week, suggesting that the percentage of these sessions focusing on child health during the year is low. This suggests that UK Foundation Programme administrators should consider including more content related to child and adolescent health in the future.

Confidence as a medical practitioner is directly related to experience, and as it is often said, children are not simply small adults [11]. Clearly, there are huge differences in paediatric versus adult medicine in terms of paediatric anatomy, physiology, pharmacology, ethics and the efficacy of medical and psychosocial interventions. Existing studies have shown that non-paediatric specialists often feel uncomfortable or have incomplete knowledge regarding paediatric care. For example, a survey of 166 GP trainees found that 40.4% reported receiving no teaching on paediatric prescribing during their undergraduate training, and 30.1% reported receiving no equivalent teaching as a part of their postgraduate training [12]. A survey of 346 doctors in the UK, primarily non-paediatricians, found that self-rated confidence in managing paediatric musculoskeletal problems was low, and only 52% recalled ever receiving paediatric musculoskeletal teaching, compared to 92% who recalled receiving adult musculoskeletal teaching [13]. In a survey of UK obstetrics and gynaecology trainees, 24.7% were unaware whether there was a paediatric obstetric and gynaecology clinic at their own hospitals [14]. In our study, trainees working in paediatric posts reported attending significantly more paediatrics teaching, as did those with an interest in paediatrics. Therefore, it is vital to integrate teaching on paediatric and child health within the core Foundation curriculum as it remains the last opportunity to provide broad education to all trainee doctors, whether they become specialists or generalists later on. Previous qualitative studies have described trainees receiving mandatory teaching that was not always perceived as relevant to clinical learning and development, instead focusing on 'audit, ethics, law, protocols' [15]. Although these areas are relevant and important, our findings suggest that it will be vital to consider whether the UK Foundation Programme curriculum needs further adjustment, to determine whether there is sufficient coverage of child health, among other areas.

Among other outcomes, it is concerning that 15% of the respondents reported not receiving teaching in child protection/safeguarding during F1/F2 (including e-learning) even though this is a mandatory requirement for ARCPs. It is possible that some participants’ recollections may be unreliable, but the lack of recall of attending teaching may accompany a lack of recall of safeguarding principles. Safeguarding is a shared responsibility for all staff but especially frontline staff who will regularly be involved in the care of children. It is vital to ensure that all doctors are aware of their responsibilities and have up-to-date knowledge on child protection processes, especially given the disruption in education and training that occurred during the COVID-19 pandemic, which may have impacted knowledge and exposure [16]. In the context of growing clinical pressures, new and innovative technologies in medical education, such as artificial intelligence, could offer new approaches to teaching in complex scenarios [17]. 

Study limitations

There are a number of limitations to this study, including recall bias. This study is likely to include a greater-than-average proportion of Foundation doctors with an interest in paediatrics, given that participation was optional and respondents with a prior interest may be more likely to participate in a survey related to paediatrics, so medians for the overall cohort may be an overestimate. In addition, the survey was distributed in the final two months of the academic year, so it is possible that for some F1 doctors, only 10 months’ worth of teaching would have been considered when answering this survey. This may have led to a slight underestimation in the amount of teaching in paediatrics offered for these trainees. Certain deaneries were better represented than others, so this study is not representative of practice in all areas. As an anonymous survey, individual identities of respondents could not be verified. Finally, only a very small percentage of the total cohort of Foundation doctors across the UK (which is around 16,000 at any one time) responded to the survey. Although, to our knowledge, this survey represents the largest quantification to date of Foundation doctor teaching in paediatrics, this remains a limitation. The ultimate response rate cannot be estimated because it is unknown how many educational departments ultimately distributed the survey.

## Conclusions

This study is the first to formally quantify the number of training hours in paediatrics received by Foundation doctors per year. From over 200 respondents, we find that this is low (a median of two hours). This is notable, considering that most doctors will care for children on a routine basis as a part of their later careers if working in areas, such as general practice, emergency medicine, surgery or paediatrics itself. We recommend targeted adaptation of teaching programmes to provide more training on key areas of uncertainty to ensure continued high-quality care for children and young people in the coming years.
